# Perspectives on the ethical concerns and justifications of the 2006 Centers for Disease Control and Prevention HIV testing recommendations

**DOI:** 10.1186/1472-6939-12-24

**Published:** 2011-12-16

**Authors:** Michael J Waxman, Roland C Merchant, M Teresa Celada, Melissa A Clark

**Affiliations:** 1 Department of Emergency Medicine, Alpert Medical School of Brown University, 593 Eddy Street, Providence, Rhode Island, 02912, USA; 2 Department of Community Health, Alpert Medical School of Brown University and Program in Public Health, 121 S Main Street, Providence, Rhode Island, 02912, USA; 3 Department of Philosophy, Wheaton College, 26 E. Main Street, Norton, Massachusetts, 02766, USA

## Abstract

**Background:**

In 2006, the Centers for Disease Control and Prevention (CDC) recommended three changes to HIV testing methods in US healthcare settings: (1) an opt-out approach, (2) removal of separate signed consent, and (3) optional HIV prevention counseling. These recommendations led to a public debate about their moral acceptability.

**Methods:**

We interviewed 25 members from the fields of US HIV advocacy, care, policy, and research about the ethical merits and demerits of the three changes to HIV testing methods. We performed a qualitative analysis of the participant responses in the interviews and summarized the major themes.

**Results:**

In general, arguments in favor of the methods were based upon their ultimate contribution to increasing HIV testing and permitting the consequent benefits of identifying those who are HIV infected and linking them to further care.

**Conclusions:**

The prevailing theme of ethical concern focused on suspicions that the methods might not be properly implemented, and that further safeguards might be needed.

## Background

The 2006 US Centers for Disease Control and Prevention (CDC) recommendations for HIV testing in healthcare settings contain three distinct changes to HIV testing methods from prior recommendations designed to streamline and encourage widespread testing: (1) use of an opt-out HIV approach to introduce HIV screening and diagnostic testing to patients, (2) use of the general medical consent for care instead of separate signed consent for HIV testing, and (3) removal of a requirement to conduct HIV prevention counseling at the time of HIV testing [1]. The 2006 CDC recommendations received a mixed and heated public reaction from academicians, advocates, clinicians, government officials, and researchers [2-34]. Some offered moral praise, others moral condemnation.

On the one hand, some opponents cited the possibility for costs or bad consequences that might follow implementation of the recommendations, while some supporters cited the potential for good consequences. On the other hand, some opponents claimed that implementation of the recommendations might violate patients' rights, while some supporters denied this. A review of the literature on the moral evaluation of the recommendations showed that commentators singled out harms or benefits without balancing the set of harms and the set of benefits [35]. However, to identify a possible advantage to the recommendations is not to justify them. Similarly, to identify a possible disadvantage or a moral concern about the recommendations is not to justify their rejection.

The objective of this research was to obtain a more systematic, balanced, and in-depth evaluation of the CDC recommendations from participants in their public debate. We conducted qualitative interviews with academicians, members of advocacy groups, clinicians, policymakers, and researchers who had voiced their opinions in the media and professional and lay literature in an attempt to elicit from them a systematic ethical analysis of the CDC recommendations. First, we asked respondents to consider the potential benefits and the possible risks or harms posed by the revised HIV testing strategies. Second, we asked respondents whether in their view each recommendation fulfilled or violated moral responsibilities to patients. Third, we asked respondents if they thought each recommendation was respectful or a violation of patient rights. We performed a qualitative analysis of these participant responses and provide in this manuscript an accounting of the predominant themes. Our aim was to inform the continuing debate on the implementation of the three CDC-recommended HIV testing methods, especially in light of CDC's plan to release new recommendations for HIV testing in non-healthcare settings [36]. It might be true that the 2006 CDC HIV testing recommendations can be acceptable to all parties with minor modifications. However, this conclusion can only be reached after the detail and nuances of the ethical arguments---both for and against---are explored in a balanced and systematic fashion.

## Methods

### Study design

This manuscript reports on a qualitative analysis of responses to semi-structured interviews of 25 members from the fields of US HIV advocacy, clinical or social care, policy, or research who had commented on the 2006 CDC HIV testing recommendations in the media or lay or professional literature. The authors' institutional review board approved this study.

### Study participant population

In August 2007, we performed a search of MEDLINE, Philosopher's Index, SocIndex, the internet, and medical and public health journal websites for all articles, commentaries, editorials, press releases, publications, research, statements, etc. about the 2006 CDC HIV testing recommendations [35]. From this search, 164 documents or websites met this criterion. From these, 55 authors or persons quoted were identified, and consequently formed the group of potential study participants. US government officials were excluded as potential study participants because of potential conflicts of interest from their criticizing or praising the CDC recommendations.

An investigator who did not conduct the semi-structured interviews sorted the 55 potential study participants first by whether or not they, according to their comments, supported or did not support the CDC revised recommendations, and second by their primary occupation. This sorting yielded five strata of participants: supportive advocates (n = 11), concerned advocates (n = 11), supportive academicians/clinicians/researchers (n = 15), concerned academicians/clinicians/researchers (n = 9), and government (non-federal) officials (n = 9). Using these strata, a list of potential participants was generated.

In accordance with recommendations for conducting qualitative research, we chose an a priori sample size of 25 participants (five from each of the five strata) [37-39]. A research assistant, who was not involved in the interviewing process, contacted and invited potential participants by email, letter, and telephone to participate in a telephone interview. When extending the invitations to potential participants, the research assistant attempted to achieve a gender-balanced and geographically-diverse participant sample. Invitations were extended to potential participants until each stratum was filled with completed interviews. No incentives were provided to participants.

### Survey development

The study authors developed a brief survey for the semi-structured interviews. The survey contained questions about three recommended changes to HIV testing methods germane to this analysis: (1) using an opt-out approach to HIV testing, (2) replacing separate signed consent with general medical consent for HIV testing, and (3) making HIV prevention/risk reduction counseling optional instead of mandatory (or uncoupling prevention/risk counseling from testing). For each of these three topic areas, the survey contained open-ended questions that asked respondents for their opinions on the topics according to four ethical domains: (1) the benefits of the change in HIV testing methods; (2) the risks or harms of the change; (3) how the change in testing methods fulfills or violates ethical responsibilities of a health care provider to administer appropriate medical care to patients; and (4) how the change in testing methods respects or violates patient rights. By respecting or violating patient rights, we asked participants to consider how the ethical domain under discussion allow patients to receive appropriate health care yet still protect their civil liberties to choose which medical practices will be performed on them. These four ethical domains were chosen by our multidisciplinary group of investigators (from the fields of clinical medicine, community health, epidemiology, philosophy-bioethics, and survey research methodology) from a review of the 164 documents and websites yielded by the literature searches. These four domains reflect the most common approaches to the ethical evaluation of policies: consequentialist and rights approaches. The survey used was informed by the two distinct kinds of moral frameworks prevalent in the expressed responses to the CDC recommendations: a consequentialist framework and a rights framework. Of the four familiar principles of bioethics beneficence and non-maleficence are thought to be grounded in a consequentialist framework, while duties to respect autonomy and duties of justice are often correlated with a rights-based approach to morality [40]. A copy of the survey relevant to the analysis is provided with the manuscript.

### Survey administration

Participants received a copy of the 2006 CDC HIV testing recommendations and a brief synopsis of the study and study topics prior to the telephone interviews. Two of the study authors conducted the interviews; each author conducted approximately half of the interviews. The interviewers were blinded to participant strata and participants' previous comments in the lay and medical literature. All interviews were conducted via telephone from October 2007 through August 2008.

At the start of each interview, each participant was provided with a description of the nature of the study, the topics under discussion, the questions that would be asked, and definitions of any terms used in the interviews. Participants were asked to confine their responses to ethical considerations about each HIV testing method, as opposed to their practical implementation. Participants were prompted as necessary by the interviewers to focus their answers on the specific HIV testing method under discussion, and reminded to avoid commenting on the remaining HIV testing methods or the recommendations as a whole. Participants giving short answers to the questions were prompted to elaborate. Interviewers did not otherwise provide any commentary or feedback to the participants. All interviews were audio-recorded and later transcribed and de-identified by a research assistant who was not involved in the interviews.

### Analysis

A qualitative content analysis was performed on the de-identified transcripts without regard to participant strata. A quantitative analysis of the responses was not performed, due to the sample size chosen, methods of the survey, purposive stratification of respondents, and goals of the planned analysis. The transcribed survey responses were reviewed and coded by the two interviewers. The two interviewers identified themes, sub-themes, and sub-sub-themes implicit in the participant responses. Two secondary reviewers, who had not conducted the interviewers, independently reviewed separate random samples of 50% of the transcripts for accuracy and thoroughness of the data extraction and selection of themes, sub-themes, and sub-sub-themes. Afterwards, the primary and secondary reviewers discussed their findings; inconsistencies were discussed and reconciled.

## Results and Discussion

### Overarching and recurring themes and overall observations

A common overarching theme that permeated the interviews was that any ethical considerations of the merits or demerits of the HIV testing methods depend heavily on how they will be implemented. As one participant said, "the devil is in the details." Many participants stated that if the HIV testing methods were implemented in a fashion that patients were aware they were being tested, had the opportunity to refuse testing, were given adequate pre-test information to make an informed decision about being tested, and had an opportunity to engage in further discussion with their healthcare provider, then the testing methods fulfilled ethical responsibilities to patients and respected patient rights. Conversely, if the HIV testing methods were implemented in a way that patients were being tested without their knowledge, or without consent, or without knowing the reasons to be tested and the consequences of being tested (and the consequences of the test results), or patients were not given an opportunity to discuss the test further with their medical provider, then the HIV testing methods would not fulfill ethical responsibilities to patients and violated patients' rights. As expressed by one participant in regards to using the opt-out approach, "It depends, and this is actually one of the biggest ambiguities in the recommendations. I think as a practical manner, opt-out testing in and of itself does not necessarily violate patients' rights. I think it heightens the risk for patients to have their rights violated, but I do believe that opt-out testing can be conducted ethically and in a way that protects patients decision-making and informed consent rights, although...I think there is a heightened risk that those protections won't occur."

Another recurring theme was that many participants viewed the HIV testing methods as a set that could be implemented only as a bundle, as opposed to individual testing methods that could be used individually or jointly as needed. This perspective is understandable given the intent of CDC that these methods be implemented as a whole. As a consequence of this view, respondents who voiced objections to one of the three testing methods sometimes would conclude that another method or the entire set of CDC's recommendations was morally problematic. This phenomenon was evident when participants often gave similar justifications or concerns for each of the three HIV testing methods.

Participants also expressed different underlying opinions -which were reflected in their responses to the questions- as to whether or not HIV testing ought to be treated differently than testing for other medical conditions. Those participants who believed that HIV testing should be treated differently than other types of medical tests generally believed that more safeguards should be administered than which the CDC testing methods recommend. As one participant commented, "You're doing a utilitarian calculus, which goes against patient autonomy in favor of trying to identify as many HIV positive patients as possible, and that creates mistrust in the patient-physician relationship." On the other hand, those participants who believed that testing for HIV should be treated similar to testing for other treatable medical conditions generally believed that additional safeguards would only impede patients from receiving what they viewed to be a potentially lifesaving screening exam. One participant said, "I think it fulfills our ethical responsibility to look for a disease that is ultimately treatable and treatable well if you find it early and devastating if it is missed."

### Opt-out approach to HIV testing (Table 1)

#### Benefits of the opt-out approach

The benefits of the opt-out approach can be summarized along three main themes: opt-out testing (1) improves the HIV testing process by overcoming barriers to testing through making HIV testing more convenient for the patients when receiving other types of medical care; making HIV testing more of a standard part of medical care; enabling testing in patients who otherwise might be reluctant to broach the topic of HIV testing; and streamlining the HIV testing process, which makes it easier for providers to introduce HIV testing and routinize HIV screening; (2) has beneficial consequences to individuals, public health, and society by allowing more people to know their status, access life-saving treatment, and protect loved ones from society; by permitting HIV-infected individuals to learn their status earlier in the disease course, which benefits the individual and helps reduce transmission; and by decreasing stigma and HIV exceptionalism; and (3) has beneficial downstream consequences, such as increasing the number of people who know their status, giving HIV-infected patients an opportunity to start lifesaving antiretroviral therapy earlier in their disease course, and providing an opportunity for those infected with HIV to protect others.

**Table 1:** Themes of the opt-out approach to HIV testing

*Benefits*	*Risks or Harms*
***Opt-out HIV testing...*** ***Theme***: Improves the testing process ***Sub-theme***: Streamlines the testing process ***Sub-theme***: Overcomes barriers ***Sub-subtheme***: Overcomes barriers to the individual being tested ***Sub-subtheme***: Overcomes barriers to the provider conducting testing ***Theme***: Has beneficial consequences ***Sub-theme***: Beneficial consequences to public health ***Sub-theme***: Beneficial consequences to individuals ***Sub-theme***: Beneficial consequences to society ***Theme***: Leads to increased testing, which has beneficial downstream effects	***Theme: ***There are risks and harms inherent in the method or process of opt-out HIV testing ***Theme: ***There are associated problems when using opt-out HIV testing ***Sub-theme***: Opt-out testing does not provide adequate consent ***Sub-theme***: Opt-out testing does not provide adequate pre-test information ***Sub-theme: ***Opt-out testing will lead to increased testing, which can lead to an increased number of false positives or testing people who do not want to be tested ***Theme: ***There are conditions or situations when opt-out HIV testing presents harms or risks

***How does opt-out HIV testing*** ***fulfill responsibilities to patients?*** ***Opt-out HIV testing...*** ***Theme***: Leads to increased HIV testing, which has downstream effects ***Theme***: Promotes HIV screening in clinicians ***Theme***: Changes a paradigm of HIV testing ***Theme***: Leads to increased testing, which has downstream effects ***Theme***: Promotes patient health ***Theme***: Overcomes patient barriers to testing	***How does opt-out HIV testing*** ***violate responsibilities to patients?*** ***Opt-out HIV testing...*** ***Theme: ***Opt-out testing does not allow for adequate information delivery ***Theme: ***Opt-out testing changes the beneficiary of testing from the patient to public health or society ***Theme: ***Opt-out testing negatively affects patient trust

***How does opt-out HIV testing*** ***respect patients' rights?*** ***Opt-out HIV testing...*** ***Theme***: Provides an adequate process for patients to refuse testing ***Theme***: Leads to increased testing, which has positive downstream affects on individuals ***Theme***: Serves individuals and pubic health needs	***How does opt-out HIV testing*** ***violate patients' rights?*** ***Opt-out HIV testing...*** ***Theme***: Will likely be poorly implemented, which will violate patients' rights ***Theme***: Does not provide for an adequate process for HIV testing ***Theme***: Compromises individual patient needs ***Theme***: Limits patients' options to make their own decisions

#### Risks or harms of the opt-out approach

For the risks or harms for the opt-out approach, the first theme centered on concerns inherent in the method or process of the opt-out approach, such that test recipients would not understand that they are being tested for HIV, that the opt-out approach is by its nature is coercive by limiting patient options and decision-making, and that the opt-out approach is not patient centered. Respondents were also concerned that the opt-out approach must be performed with no separate signed consent for HIV testing. Therefore, the opt-out approach limits the consent process and that patients might be tested with limited or no informed consent. In addition, some respondents were concerned that the opt-out approach might also be practiced with limited or no pre-test information. The second theme concerned perceived associated problems and negative downstream consequences of the opt-out approach, particularly related to increased testing. Examples cited included the problems of initiating, securing and following through with referrals for treatment among patients diagnosed with an HIV infection who were not psychologically or emotionally prepared for their results or who may not have the resources to find medical care, and the problems associated with false-positive test results. The third theme entailed specific conditions or situations where the opt-out HIV approach might be practiced in an unethical fashion. For example, vulnerable populations - such as those with lower health literacy, those who do not speak English, or intoxicated patients - might not be fully aware they are being tested. One respondent stated with regards to those individuals who may not understand that they are being tested, "There are issues around language barriers, people who may be under some chemical influence, people who are not in a state of being ready to respond to what is being offered to them and not really understanding what is happening in the health care setting..." Furthermore, in busy clinical settings, the opt-out approach might give the clinician an excuse to rush the procedure such that patients do not have an opportunity to decline testing. Finally, patients might feel that saying no to their clinician would negatively impact their relationship with their clinician.

#### Fulfilling responsibilities to patients and respecting patients rights with the opt-out approach

In terms of fulfilling ethical responsibilities to patients and respecting patient rights, the themes included a view that the opt-out approach was a means of promoting and enabling clinicians to perform HIV testing through routinizing the testing process and reducing the barriers associated with HIV testing, which further enables clinicians to promote the health of their patients. Similarly, the opt-out approach was viewed as a way to enable patients to be tested who would otherwise be reluctant to ask for HIV testing, thereby giving individuals an opportunity to improve their health. Some participants stated that the opt-out approach changes the paradigm of HIV testing from a "special" or "scary" test to a routine preventive health measure and reduced HIV exceptionalism. Some stated that the opt-out approach respected patients' rights because it provided an adequate process for patients to refuse testing and gave them an opportunity to gain access to a beneficial test.

#### Violating responsibilities to patients and patients' rights with the opt-out approach

The opt-out approach was viewed by some respondents as violating responsibilities to patients because it might shift the beneficiary of testing from the individual to society. Therefore, the opt-out approach could create a barrier between the patient and provider and negatively affect patient trust. The opt-out approach was viewed as not providing adequate delivery of information to patients, especially in regards to the fact that they were being tested for HIV, and that the risks and benefits of HIV testing were not adequately conveyed, particularly for special populations, such those who speak other languages, are developmentally disabled, intoxicated, etc. Some respondents also commented that the opt-out approach would be poorly implemented, and consequently would not permit adequate pretest information to be delivered or provide opportunities for patients to decline testing. Finally, the opt-out approach was viewed as a means of violating patients' rights because the approach inherently limits patients' options to make their own decisions, and therefore they will not have a true opportunity to decline testing.

#### Removing the separate, signed consent requirement (Table 2)

Some respondents thought the moral acceptability of the removal of the requirement for a separate signed consent was conditional on a number of factors. No separate signed consent was often considered ethical if the clinician ensured that patients were aware that they were being tested for HIV, and if they provided opportunity for discussion. Some respondents further specified that no separate signed consent would be ethical if the patient provided verbal consent for testing. Others thought no separate signed consent for HIV testing was necessary if the general consent document made explicit mention of HIV testing.

**Table 2:** Themes of no separate signed consent

*Benefits*	*Risks or Harms*
***Theme: ***Leads to better utilization of HIV testing ***Theme: ***Positively affects the HIV testing process ***Sub-theme: ***Streamlines ***Sub-theme: ***Overcomes barriers ***Theme: ***Positively affects the perception of HIV and HIV testing ***Theme: ***Leads to increased testing, which has positive downstream effects	***Theme: ***When specific signed consent is eliminated there is a loss of a safeguard to HIV testing ***Sub-theme***: Inadequate transfer of information ***Sub-theme***: Lack of truly informed consent ***Sub-subtheme: ***May bring harm to specific populations ***Sub-subtheme: ***May lead to negative downstream consequences ***Sub-theme: ***Passivity in the consent process ***Sub-theme***: Loss of legal protection for providers ***Theme: ***No specific signed consent harms the trust patients have in their providers and the medicals system ***Theme: ***No specific signed consent de-emphasizes individual healthcare needs

***How does no separate signed consent fulfill responsibilities to patients?*** ***Theme***: General consent for medical care might be considered an equivalent consent mechanism to separate signed consent ***Theme***: Reduces barriers to HIV testing ***Theme***: Leads to increased testing, which has positive downstream effects ***Theme***: "Mainstreams" HIV testing ***Theme***: Improves the HIV testing experience for patients	***How does no separate signed consent violate responsibilities to patients?*** ***Theme: ***Does not provide for adequate delivery of information ***Theme: ***Does not acknowledge that HIV testing is different than testing for other diseases ***Theme: ***Negatively affects patient trust

***How does no separate signed consent*** ***respect patients' rights?*** ***Theme***: Incorporates a process into good, standard clinical care ***Theme***: Leads to increased testing, which has positive downstream effects ***Theme***: Reduces stigma	***How does no separate signed consent*** ***violate patients' rights?*** ***Theme: ***Eliminates safeguards in obtaining proper informed consent ***Sub-theme***: Does not allow for adequate assessment of patient comprehension ***Sub-theme***: Might lead to improper procedures, which could have negative consequences ***Theme: ***Compromises individual patient needs ***Theme: ***Does not respect patient autonomy ***Theme: ***May lead to paternalistic and poor clinician actions ***Theme: ***May inadvertently reduce patients' access to care

#### Benefits of removing the separate signed consent requirement

The first theme regarding the benefits of removing the separate signed consent requirement was that no separate signed consent improves the HIV testing process by eliminating a cumbersome consent process and decreasing the onerous paperwork associated with HIV testing. The second theme was that the removal of separate signed consent positively affects the perception of HIV and HIV testing by making HIV testing seem to be a standard part of medical care, reducing the impression that testing for HIV is more dangerous than testing for other treatable medical conditions, destigmatizing HIV, and reducing the impression to patients that they are being profiled or segregated when performing HIV testing. As one respondent stated, "not requiring additional consent would mean that everyone is eligible to receive an HIV test without any additional burden of obtaining any separate consent for HIV testing. So again, it normalizes the testing and makes it similar to other types of medical testing that is done in a healthcare setting such as cholesterol screening, such as cancer screening."

The third theme was that separate signed consent would increase the number of patients tested for HIV and, therefore, would have positive downstream consequences, as discussed previously. The fourth theme underscored the belief that HIV testing is a very powerful tool to help individuals and populations, but that in its current form is not being fully utilized. By removing the cumbersome consent process and paperwork, clinicians will be more apt to conduct HIV testing and incorporate HIV testing into standard medical care. In addition, removal of separate signed consent facilitates testing of those patients who do not fully understand the benefits of HIV testing.

#### Risks or harms of removing the separate signed consent requirement

The first of three themes on the risks of harms of removing the separate signed consent requirement was that a separate signed consent for HIV testing serves as a necessary safeguard, which ensures that patients were only being tested with knowledge and consent. Some participants were concerned that no separate signed consent would lead to a passive or cavalier disregard for the consent process, loss of legal protections for patients, inadequate assessment of a patient's comprehension that they are being tested, inadequate transfer of information, and lack of truly informed consent. Some respondents also stated that absence of a separate signed consent might be particularly harmful for vulnerable populations and for those who might not be prepared for the potential negative consequences - such as knowledge of the legal implications of being HIV-infected, transmitting HIV to others, or to their insurance. One respondent stated, "... the social risks of HIV testing are often not well known, so, individuals who don't know their HIV status or may not know the risks of testing HIV positive to their health care coverage, to their status as a resident or citizen, to their heightened risk for work place or housing discrimination, the family or friend being ostracized, the stigma of discrimination. Those are real risks that exist today and I believe that would qualify as needing the definition for informed consent..." The second theme centered on harm to the trust patients place in their medical providers and healthcare systems. Some patients may perceive HIV testing without separate signed consent as an attempt to test people without their knowledge or permission. Therefore, these patients may feel uncomfortable with the new testing process or might avoid presenting for healthcare because of a fear of being tested for HIV. The third theme was that good medical practice focuses on individual patients' needs, and that no separate signed consent de-emphasizes this concept. Some respondents stated that testing with no separate signed consent might be perceived as paternalistic and violating patient autonomy by prioritizing societal over personal needs.

#### Fulfilling responsibilities to patients and respecting patients' rights by removing the separate signed consent requirement

Several themes emerged on how the removal of the separate signed consent requirement might fulfill ethical obligations to patients and respect patents' rights. Some participants stated that removal of this requirement fulfilled responsibilities to patients by reducing barriers, which had prevented patients from receiving a beneficial test. Reducing barriers would lead to more testing and the positive downstream effects of increased testing. Some participants also stated that eliminating separate signed consent also fulfilled responsibilities to patients because it "mainstreamed" HIV testing, making it similar to other types of medical tests and more feasible to include HIV testing in a routine battery of tests. Eliminating separate signed consent was also said to improve the HIV testing experience for patients, both by "mainstreaming" HIV testing and by making the HIV testing process less onerous. Some participants stated that no written consent respected patients' rights in that it facilitated good, standard clinical care for patients.

#### Violating responsibilities to patients and patients' rights by removing the separate signed consent requirement

In terms of how removal of the separate signed consent requirement might violate patients' rights, the themes involved concerns of the elimination of a necessary safeguard in HIV testing and an assessment of patient comprehension. Some participants commented that the intent of eliminating separate signed consent is to avoid an active discussion about HIV testing with patients, which undermines patients' autonomy. Further, the themes expressed on this topic were under the premise that if or when patients are tested without their knowledge or permission, this action would violate responsibilities to patients and patients' rights, might erode patients' confidence in their healthcare providers, and compromise patient autonomy and individual patients' needs. This lack of confidence might further dissuade patients - especially those at highest risk - from seeking medical attention.

### Uncoupling HIV prevention counseling from HIV testing (Table 3)

#### Benefits of uncoupling HIV prevention counseling from HIV testing

The benefits or ethical justifications for uncoupling HIV prevention counseling from HIV testing were grouped into the following themes. One theme was that this uncoupling would streamline the HIV testing process by removing a step that might not be necessary in all circumstances. Another theme that emerged was that uncoupling HIV counseling would increase the number of individuals tested for HIV and, therefore, has important downstream consequences, as discussed previously. Another theme was that this uncoupling utilizes resources more efficiently at the time of HIV testing, especially for low-risk patients, repeat testers, and those knowledgeable about HIV; and reduces the need for training for prevention counseling and the costs of testing, and was a better use of limited staff. With fewer resources needed, healthcare providers could expand access to HIV testing. Another theme which emerged was that uncoupling HIV prevention counseling deregulates HIV testing. Respondents stated that uncoupling removes a requirement that is ignored anyway; permits clinicians to individualize counseling needs for patients; and, protects patients' right to privacy by not making them divulge information about their risk behaviors. The final theme was that uncoupling makes HIV testing similar to other types of medical tests which improves and destigmatizes the perception of HIV and HIV testing.

**Table 3:** Themes of uncoupling prevention counseling from HIV testing

*Benefits*	*Risks or Harms*
***Theme: ***Positively affects perception of HIV and HIV testing ***Theme: ***Expands access to HIV testing ***Theme: ***Utilizes resources more efficiently ***Theme: ***Positively affects the HIV testing process ***Sub-theme: ***Streamlines ***Sub-theme: ***Overcomes barriers ***Theme: ***Deregulates HIV testing process ***Sub-theme: ***Benefits patients ***Sub-theme: ***Benefits providers ***Theme: ***Leads to increased testing, which has positive downstream effects	***Theme: *Loss of educational opportunity** ***Theme: ***Negative effects on risk-taking behaviors ***Theme: ***Discourages good clinical practice ***Theme: ***Fails to address patient needs ***Theme: ***Deleteriously affects other elements in the HIV testing process

***How does uncoupled prevention counseling fulfill responsibilities to patients?*** ***Theme***: Expands access to HIV testing ***Theme***: Deregulates HIV testing ***Theme***: Leads to increased testing, which has positive downstream effects	***How does uncoupled prevention counseling violate responsibilities to patients?*** ***Theme***: Leads to a loss of an educational opportunity ***Theme***: Does not acknowledge that HIV testing is different than testing for other diseases ***Theme***: May have negative effects on risk-taking behavior

***How does uncoupled prevention counseling*** ***respect patients' rights?*** ***Theme***: Protects patients' right to privacy ***Theme***: Respects patient autonomy ***Theme***: Respects individual patient needs ***Theme***: Leads to increased testing, which has positive downstream effects ***Theme***: Deregulates HIV testing	***How does uncoupled prevention counseling*** ***violate patients' rights?*** ***Theme***: Limits access to medical care

#### Risks or harms of uncoupling HIV prevention counseling from HIV testing

The first theme of the risks or harms of uncoupling prevention counseling was that this action fails to address patients' needs. Several respondents stated that HIV testing is an ideal "teachable moment" to perform prevention counseling, that counseling was a crucial part of the HIV testing process, and discourages good clinical practice. Some expressed concerns that uncoupling prevention counseling might encourage HIV risk-taking behaviors. Some respondents expressed beliefs that if prevention counseling were optional, risk assessments might never be performed; and those patients who would benefit from it might not be identified. The second theme underscored the perspective that all three HIV testing strategies - opt-out testing, no separate signed consent, and uncoupled prevention counseling - are linked and interdependent. As such, mandatory prevention counseling at the time of testing ensures that patients are educated about HIV testing and understand the consequences of the test. Uncoupling this component removes another safeguard. The final theme was that HIV is different from other medical conditions and deserves to be treated exceptionally. Therefore, HIV testing may require additional counseling that other medical conditions do not.

#### Fulfilling responsibilities to patients and respecting patients rights by uncoupling HIV prevention counseling from HIV testing

The major theme regarding fulfilling responsibilities to patients by uncoupling HIV prevention counseling from HIV testing was that this strategy expands access to and deregulates HIV testing. These actions would then lead to increased HIV testing, which has downstream benefits to patients, as discussed previously. Participants believed that uncoupled prevention counseling respects patients' rights because, by not requiring patients to divulge their risk taking behaviors in order to get tested for HIV, it respected patients' privacy and autonomy.

#### Violating responsibilities to patients and patients rights by uncoupling HIV prevention counseling from HIV testing

In terms of violating responsibilities to patients and patients' rights, one major theme underscored a belief in the value of the "teachable moment" to counsel patients on risk taking behaviors that would be lost if the uncoupling strategy were used. In addition, uncoupling might violate patients' rights by limiting their access to better health care. Some participants envisioned that clinicians would cease to offer prevention counseling to even the patients most in need.

Several respondents stated that there were situations under which prevention counseling did not need to be coupled with HIV testing, although most added that some degree of information or counseling should be available to patients as deemed appropriate. As one respondent said, "... for some patients who are again very experienced with HIV testing, recognize ... the methods of transmission, and are coming in for regular HIV screening they may not need any kind of prevention counseling. For folks who are who are less familiar with HIV testing, in particular less familiar with HIV itself, I think that their rights would be better fulfilled by giving them more information and counseling at the time of testing." Some also noted that the uncoupling of prevention counseling from HIV testing was an "ethical trade-off" between testing more people with less counseling vs. testing fewer people with more counseling.

## Conclusions

Since the CDC promulgated their 2006 HIV testing recommendations, stakeholders in the HIV/AIDS advocacy, care, research, and policy have cited both potential ethical concerns and justifications for the 2006 CDC recommendations. Some of the concerns are theoretical and postulated, others possibly based upon the experience with conducting HIV testing and providing services to those who are being tested or who are affected by HIV/AIDS. Equally so, some of the justifications are based upon research or experience, or might reflect philosophical perspectives on how to best achieve broader acceptance and utilization of HIV testing. The objective of this study was not to weigh the merits of these views, but present them simultaneously, objectively, and without comment. Those who have concerns about the recommendations may now understand the perspectives of those who support their implementation, and vice versa. Perhaps a common ground can be reached that promotes effective HIV testing that is inline with current perspectives on medical and public health ethics. The task that remains is to, whenever possible, conduct research to determine which concerns are shown to be valid when the recommendations are implemented, and when modifications of the recommendations are needed. Likewise, if research indicates that the methods are justified by their end results and concerns are not found, then implementation can to improve identification of those who have HIV but are unaware of their infection.

As raised by some respondents, whether or not the CDC recommendations will be implemented properly is an open question. The specific concerns highlighted in the themes uncovered in this investigation indicate areas where caution might be necessary, suggest topics for which specific training and explicit safeguards might be established, and where further research is needed. We are hopeful that they may be used to instruct those who implement the testing methods so that they might be used properly, to motivate the continued expansion of HIV screening and diagnostic testing, and shape future policies on HIV testing. We are also hopeful that the ultimate good intent of the recommendations can be used as a framework for their implementation and further helpful dialogues on improving HIV testing methods.

## Competing interests

The authors declare that they have no competing interests.

## Authors' contributions

MJW participated in the conception and design of the study, the data collection and coding, and writing of the manuscript. RCM participated in the conception and design of the study, the data collection and coding, and writing of the manuscript. MTC participated in the design of the study, the coding of data, and reviewing the manuscript. MAC participated in the design of the study, the coding of data, and reviewing the manuscript. All authors read and approved the final manuscript.

## Pre-publication history

The pre-publication history for this paper can be accessed here:

http://www.biomedcentral.com/1472-6939/12/24/prepub
